# Ecopathology of Ranaviruses Infecting Amphibians

**DOI:** 10.3390/v3112351

**Published:** 2011-11-22

**Authors:** Debra Miller, Matthew Gray, Andrew Storfer

**Affiliations:** 1 Center for Wildlife Health, University of Tennessee, Knoxville, TN 37996, USA; E-Mail: mgray11@utk.edu; 2 Department of Biomedical and Diagnostic Sciences, College of Veterinary Medicine, University of Tennessee, Knoxville, TN 37996, USA; 3 School of Biological Sciences, Washington State University, Pullman, WA 99164, USA; E-Mail: astorfer@wsu.edu

**Keywords:** amphibian declines, anthropogenic stressors, emerging pathogen, histopathology, *Iridoviridae*, pathogen pollution, *Ranavirus*, subclinical infection, transmission

## Abstract

Ranaviruses are capable of infecting amphibians from at least 14 families and over 70 individual species. Ranaviruses infect multiple cell types, often culminating in organ necrosis and massive hemorrhaging. Subclinical infections have been documented, although their role in ranavirus persistence and emergence remains unclear. Water is an effective transmission medium for ranaviruses, and survival outside the host may be for significant duration. In aquatic communities, amphibians, reptiles and fish may serve as reservoirs. Controlled studies have shown that susceptibility to ranavirus infection and disease varies among amphibian species and developmental stages, and likely is impacted by host-pathogen coevolution, as well as, exogenous environmental factors. Field studies have demonstrated that the likelihood of epizootics is increased in areas of cattle grazing, where aquatic vegetation is sparse and water quality is poor. Translocation of infected amphibians through commercial trade (e.g., food, fish bait, pet industry) contributes to the spread of ranaviruses. Such introductions may be of particular concern, as several studies report that ranaviruses isolated from ranaculture, aquaculture, and bait facilities have greater virulence (*i.e.*, ability to cause disease) than wild-type isolates. Future investigations should focus on the genetic basis for pathogen virulence and host susceptibility, ecological and anthropogenic mechanisms contributing to emergence, and vaccine development for use in captive populations and species reintroduction programs.

## 1. Global Impact

### 1.1. Distribution of Ranaviruses in Amphibians

Ranaviruses are emerging pathogens that are known to have caused amphibian die-offs on five continents (see Table 1) [1]. The greatest number of reported mortality events has been in North America and Europe, resulting in population declines in several cases [2–6]. Ranaviruses are known to infect at least 72 amphibian species in 14 families (see Table 1). Of the published reports, the majority of cases have been in the family, *Ranidae*. Susceptibility to ranavirus infection varies widely among species [7,8]. Of 19 North American species tested, wood frog (*Lithobates sylvaticus*) and gopher frog (*L. capito*) were the most susceptible [9]. Eastern spadefoot toads (*Scaphiopus holbrookii*) are also highly susceptible to ranaviruses [10]. Rare amphibian species that are known to experience mortality from ranaviruses include the gopher frog, mountain yellow-legged frog (*Rana muscosa*), California red-legged frog (*R. draytonii*), western toad (*Anaxyrus boreas boreas*), and Chinese giant salamander (*Andrias davidianus*, see Table 1). Given the global distribution of ranaviruses and their capability to cause disease in numerous anuran and caudate species, this group of pathogens represents a significant risk to global amphibian populations.

### 1.2. Species Susceptibility

Susceptibility to ranaviruses varies among populations within species and across phylogenetic lineages [9,34]. Among populations, the amount of genetic variability appears to be correlated with ranavirus susceptibility [83,84]. For example, inbred African clawed frogs (*Xenopus laevis*) are more susceptible to ranavirus infections than outbred lines [85]. In the wild, variation in population-level susceptibility may be related to adaptation of host immune evasion genes to local ranavirus strains [86]. Thus, the likelihood of a species being infected by ranaviruses over evolutionary time may be a significant driver affecting susceptibility, which might be impacted by life history characteristics. Amphibian species in North America that develop faster as larvae, have restricted distributions, or inhabit semi-permanent breeding sites tend to be more susceptible than slow developing, widespread species that live in temporary wetlands [9]. The geographic location of populations may also factor into host susceptibility. Watersheds located at higher elevations have been associated with increased probability of ranavirus infection and epizootics in some North American amphibian populations [64,87]. Lastly, environmental conditions such as ambient temperature or pesticides have been associated with increased prevalence of ranavirus infection and disease [27,66,88,89].

The susceptibility to ranaviruses differs among amphibian developmental stages, even in the same species [1]. The adult stage tends to be the least susceptible, probably owing to more competent immune function compared to pre-metamorphic stages [90]. However, die-offs of adult frog and newt species in the wild have been reported in Europe [5,6,27,91]. In captivity, adult mortality from ranaviral disease is common [59,60,68,92], and may be a consequence of increased transmission associated with high host density, elevated viral titers, or stress induced from the unnatural environment [93].

The classical model of pre-adult amphibian immune function based on the African clawed frog predicts that susceptibility to ranavirus should be greatest during the egg, hatchling and metamorphosis stages due to early development or down regulation of the immune system, and lowest during the larval stage [94–96]. High susceptibility to ranavirus during metamorphosis has been documented in laboratory experiments (e.g., [97,98]), and several die-offs in the wild have occurred during metamorphosis [3,40,49]. Of seven North American species tested, metamorphosis was the most susceptible stage for three species—all in the family *Ranidae* [10]. The eastern spadefoot toad was most susceptible during the hatchling stage, perhaps due to delayed immune system development associated with rapid growth [10]. Rapid larval growth also may be an adaptation to reduce the likelihood of ranavirus exposure in the aquatic environment, where transmission is efficient. For example, wood frog tadpoles that were exposed to ranavirus experienced increased developmental rate compared to control tadpoles [98]. Eggs appear to be the least susceptible developmental stage [10]. Amphibian embryos are susceptible to ranavirus [36,99], thus the mucopolysaccharide/mucoprotein capsule coating the surface of the egg or the vitelline membrane around the embryo may afford protection from the virus [10]. These results emphasize that susceptibility to ranavirus depends on developmental stage, and the most susceptible stage varies among species.

## 2. Pathology

### 2.1. Clinical/Field Signs of Ranaviral Disease

**Figure 1.**
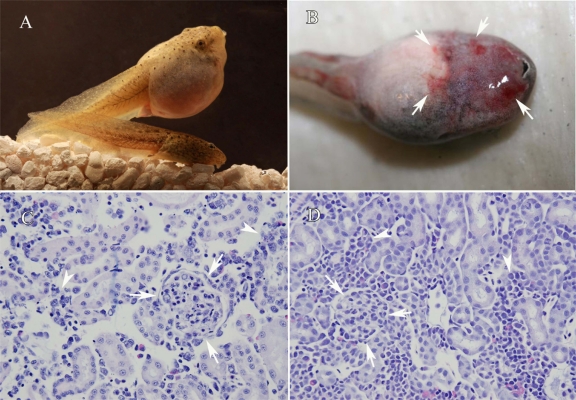
(**A**) Photo showing marked swelling of the body in a ranavirus infected American bullfrog (*Lithobates catesbianus*) tadpole (top) compared to an uninfected tadpole (bottom). (**B**) Photo showing hemorrhages (arrows) in a ranavirus infected wood frog tadpole (L. *sylvaticus*). (**C**) Photomicrograph of a mesonephros (kidney) from a ranavirus infected tadpole showing necrosis of the hematopoietic tissue (arrowheads) and degeneration and necrosis of a glomerulus (arrows). (**D**) Photomicrograph of a mesonephros (kidney) from an uninfected tadpole for comparison. Hematoxylin and eosin stain.

Mass mortality events may span days or months [56,100]; however, later deaths may be due to individuals succumbing to secondary invaders (e.g., bacterial, fungal) rather than the primary virus [51,59,98]. Mortality is often the only clinical finding reported in cases of ranaviral disease; however, erratic swimming, buoyancy problems, lethargy, and anorexia frequently occur [33,50,51,56]. In fatal cases, gross findings may include swelling of the legs and body (Figure 1A); erythema (redness) of the legs and ventrum (Figure 1B); ecchymosis (red blotches) near the vent and/or urostyle; petechiation (pinpoint hemorrhages) or ecchymosis of the skin and internal organs (especially the mesonephros (kidneys and liver); and irregular patches of discoloration on the skin. In metamorphs and adults, the gastrointestinal tract may be empty or contain minimal ingesta and the gall bladder may be enlarged, both of which are consistent with fasting. Although hemorrhages and swellings are the most common gross lesions noted in the larvae, cutaneous erosions and ulcerations are more frequently seen in adult anurans in Europe [29] and adult caudates in North America [33]. There have been additional gross lesions reported in caudates, including hemorrhages on the plantar surfaces of the feet and tail and edema in the gular region [50,56]. Jancovich *et al*. [51] further described lesions spanning 4 stages of ranaviral disease in experimentally challenged tiger salamanders (*Ambystoma tigrinum*). In Stage 1 white polypoid lesions covered approximately 10% of the body, followed by Stage 2 wherein they covered 50% of the body. By Stage 3, the polyps covered nearly 90% of the body and epidermal hemorrhages were occasionally noted. By Stage 4, hemorrhages were common and were present in internal organs, as well as in the epidermis. Lethargy, anorexia, buoyancy problems, and bloody discharge were not noted until Stage 4. Interestingly, the polypoid lesions observed by Jancovich [51] in the experimentally-challenged salamanders were not observed in field collected specimens.

### 2.2. Histopathological Findings

Ranaviral disease is characterized by systemic (throughout the body) hemorrhage and cellular necrosis, often resulting in organ failure within only a few days to 2–3 weeks of exposure [8,56,68,88,101]. Robert [96] reported that the kidney is the target organ in immunocompetent African clawed frog adults; however, other organs quickly become infected in immunocompromised individuals. Intestinal epithelial cells appear to be one of the first cell types where ranavirus can be detected as early as 3 hours post-exposure, and may be the primary route of entry into the body [102].

Although many organs may be affected, the liver, spleen and kidneys are the organs where histopathological changes are most commonly reported in fatal cases [2,59,60,68]. Renal tubular epithelium (the cells lining the tubules in the kidney) is thought to be one of the primary targets for ranaviruses; however, various studies have uncovered evidence that other cell types (e.g., fibroblasts, macrophages, ganglia, endothelial cells) are also affected [27,70,85,96,103]. Wolf *et al*. [70] reported endothelial necrosis and associated hemorrhage in vessels of multiple organs but especially the mesonephros and gastric areas. Endothelial necrosis and necrosis of the hematopoietic tissue often can be extensive (see Figures 1C,D) [104]. Similarly, Docherty *et al*. [50] found necrosis in the brain (meningese), gills, nasal tissue, adipose, trachea, muscle, bone, and periosteum. With routine staining, it is often unclear if some changes (e.g., skeletal muscle degeneration) are caused by the virus or occur secondarily (e.g., due to metabolic or endocrine insult). New techniques (e.g., *in situ* hybridization [105]; immunohistochemical staining [27,106]) are being used to identify the cell types infected by ranavirus and the associated lesions. For example, Balseiro *et al*. [27] used immunohistochemistry to detect the presence of ranavirus in various cells and found that glomeruli appear to have the most positive staining in larval toads but ganglia (particularly in the skeletal muscle) displayed the most positive staining in juvenile newts. Proof of virus presence in tissues also helps elucidate the possible routes of transmission among individuals (e.g., possible shedding from skin lesions, glandular secretions, oral mucosa, feces).

Ranaviral inclusion bodies are most often documented as variably-sized basophilic inclusions; however, they can appear as eosinophilic and this may be reflective of the stage (early *versus* late) of the disease or vary by host. Ranaviral inclusions can be difficult to confirm in areas of necrosis, as cellular debris can mimic inclusions. Jancovich *et al*. [51] additionally described intranuclear inclusion bodies in the terminal stages of ranaviral disease in tiger salamanders, but only reported observing (by electron microscopy) viral particles in the cytoplasm and intercellular spaces. Intranuclear inclusions have been seen in other species but are rare, and their composition remains unclear [104]. Interestingly, researchers in Europe have recently documented evidence of ranavirus in the nuclei of infected lizards [107]. This discovery may eventually provide answers regarding the identity of the intranuclear inclusions observed by others.

Given that secondary (opportunistic) pathogens often are found in ranaviral disease outbreaks, determining the cause of the various microscopic changes can be especially challenging. Cunningham *et al*. [2] reported hemorrhagic gastroenteritis, systemic hemorrhages, ulcerations and bacterial incursion of multiple organs of infected common frogs (*Rana temporaria*). Similar opportunistic bacterial invasions have been reported in captive anurans [59,68]. In such cases, the lesions may be primarily associated with the ranavirus, but likely also are compounded by the bacteria (see also, [2]). To date, subclinically infected individuals have rarely been secondarily infected [69,79,108].

### 2.3. Evidence of Subclinical Infections

Several field surveillance studies have reported ranavirus-infected amphibian larvae without clinical signs or histological changes (e.g., [66,75,77,109]). Gray *et al*. [66] found that 34% of American bullfrog (*L. catesbeianus*) and 30% of green frog (*L. clamitans*) tadpoles inhabiting eight farm ponds were positive for ranavirus, but at the time of collection and necropsy, they did not present with ranaviral disease. In an expanded study at 40 sites, ranavirus infection was detected in 83% of the populations; however during sampling, only one population was experiencing a die-off from ranaviral disease [55]. Other studies have reported subclinical, but infected, salamanders returning to breeding sites [109] or present in bait shops [110]. These findings validate that asymptomatic carriers of ranavirus occur in amphibian communities.

Individuals that are subclinically infected often have no significant or only nonspecific histological changes. Miller *et al*. [68] reported nonspecific histological changes (including minimal to mild lymphocytolysis, lymphoid depletion, and mild vacuolation of hepatocytes and renal tubular epithelium) in over 100 American bullfrog and 80 green frog tadpoles collected from farm ponds in Tennessee. Furthermore, a significant correlation between histopathological findings and ranavirus infection was not found in these populations [69]; however, the observed renal tubular changes may be significant given that ranaviruses are thought to target renal tubular epithelial cells [56,68,85,90,96].

Amphibians also may harbor quiescent ranavirus. Robert *et al*. [111] demonstrated that macrophages may serve as a hiding spot for *frog virus* 3 (FV3) in immunocompetent adult African clawed frogs. Morales *et al*. [112] reported that FV3 can be detected within the macrophages for up to 3 weeks post infection, well after the kidneys were shown to clear the virus (14 days). Moreover, they noted that the infected macrophages remain relatively unaffected by the virus, suggesting a possible anti-antigen (permissive) response to FV3. More recently, Robert *et al.* [102] demonstrated that immunocompetent adult African clawed frogs may shed FV3 into water, resulting in the infection of immunosuppressed cohabitants. These findings highlight the existence and potential role of carrier animals.

## 3. Ecology

### 3.1. Population Impacts

Unfortunately, few long-term data sets exist at sites with reoccurring ranavirus die-offs, hence our understanding of the impacts on amphibian populations is currently limited. Ranaviruses appear to commonly cause population fluctuations, and in some cases declines [3,113]. Populations of the common frog that experienced reoccurring die-offs from ranaviral disease in the United Kingdom declined by 81% between surveys conducted in 1996/97 and 2008 [6]. Population declines have also been shown in edible frogs (*Pelophylax esculentus*) in Denmark [22] and in tiger salamanders in western Canada [4]. Recruitment can be significantly reduced in amphibian populations experiencing ranavirus die-offs also potentially leading to declines. For example, recruitment of spotted salamanders (*A. maculatum*) and wood frogs was nearly non-existent during an 8-year study at breeding sites in North Carolina, USA, with reoccurring die-offs from ranaviral disease [48,114].

Ranaviruses have the potential to cause population extirpations. In general, epidemiological models suggest pathogens can drive hosts to extinction if density independent transmission is possible [115–117]. Three ecological conditions favor the possibility of density independent transmission in the ranavirus-host system: (1) multiple host species can be infected with different susceptibilities to infection, (2) pathogen reservoirs can exist and ranavirus persistence outside the host can be long, and (3) aggregations of breeding adults and larvae facilitate transmission at low density. Multiple lines of evidence from laboratory and field studies indicate that various amphibian species can be infected with ranavirus and some may serve as asymptomatic carriers [7–9,111,118]. Moreover, evidence is accumulating that interclass transmission of ranaviruses among amphibians, reptiles and bony fish is possible [119–124], thus increasing the possibility of multiple pathogen reservoirs. Although studies are limited, ranavirus virions may be viable outside the host for several weeks to months [125,126]. Direct transmission among larvae also can be high [83,101,127], especially for species that exhibit schooling behavior or cluster in emergent vegetation [128]. Further, many amphibian species in temperate regions are explosive breeders and behaviorally congregate in aquatic habitats at high density, despite low terrestrial adult densities [129]. Considering that ranaviruses can be transmitted easily by direct contact [127], contact rates for ranaviruses during breeding may be similar to sexually transmitted pathogens in other organisms. Collectively, these characteristics of the ranavirus system facilitate transmission at low host densities, thereby increasing the likelihood of local extirpation of hosts by this pathogen. Despite these characteristics, evidence of density independent transmission in a ranavirus-host system has not been published. Laboratory experiments generally support density dependent transmission in single-species experiments with tiger salamanders [130] and northern leopard frogs (*L. pipiens*) [41]. Importantly, even small occurrences of density independent transmission in a primarily density dependent system can result in local extirpation of a host [131].

### 3.2. Coevolution

As ranaviruses often cause frequent epizootics and in some cases population declines, these pathogens are expected to be a source of selection on their hosts [132]. Strong selection of pathogens on host populations is expected to lead to host-pathogen coevolution, for which evidence is accruing among ranavirus-host systems. For example, common frogs showed higher frequencies of particular MHC Class I alleles among previously infected individuals as compared to uninfected individuals [133]. In the tiger salamander-*Ambystoma tigrinum virus* (ATV) system, coevolution has been supported in several studies. First, there is a negative correlation between disease and cannibal frequency among salamander populations throughout Arizona, USA [134]. Although cannibals experience a performance advantage by preying on conspecifics [135], a major fitness cost is enhanced risk of ATV infection. As a result, selection should favor reduced cannibalism in populations with high ATV prevalence [134,136]. Common garden experiments suggest a genetic basis to these field observations because individuals were not plastic in the development of the cannibalistic phenotype whether ATV was present or absent, and observed differences of cannibalistic frequency in the field were replicated in the lab [137]. Second, analysis of phylogenetic concordance suggests salamander-ATV coevolution [132]. Without three host switches attributed to the movement of infected salamanders as fishing bait [52], there is complete concordance between phylogenetic trees for both salamanders and virus [132], which supports a coevolutionary history of ATV and its hosts [138]. In addition, past selection seems to have shaped spatial variation in amino acid sequences of ATV immune evasion genes among host populations, also indicative of coevolution [86].

### 3.3. Transmission

Horizontal transmission of ranavirus can occur among individuals via indirect and direct routes [1]. Transmission of ranaviruses has been documented via exposure to contaminated water [83,101,109], by direct contact with infected individuals [127] and by exposure to fomites such as virus-contaminated sediment [75]. Ingestion of infected tissue either through necrophagy or cannibalism is another effective transmission route [51,70,75,83]. The relationship between efficient water transmission and ranavirus prevalence in adults is supported by the finding that plethodontid salamanders that have strong associations with streams and aquatic larvae (e.g., *Desmognathus quadramaculatus*) have higher infection rates than terrestrial species with eggs that develop in underground burrows via direct development (e.g., *Plethodon jordani*) [64]. Exposure to infected individuals in water for 3 hours without contact can result in transmission [102], and only one second of direct contact is needed to cause infection [127]. Typically, ingestion of the virus results in faster mortality than exposure in water [8,83]. During an outbreak, it is likely that ranavirus infects hosts via multiple routes of horizontal transmission. Although vertical transmission of iridoviruses has been shown in invertebrates [139], it has not been demonstrated for ranaviruses infecting vertebrates [140]. Attempts to test for vertical transmission have yielded mixed results [36,109]. Duffus *et al*. [36] reported a ranavirus-positive larva hatched from an egg mass of a ranavirus-negative female, but that was fertilized by a ranavirus-positive male. Vertical transmission may also be possible via exposure of eggs in the cloaca during oviposition or via infected sperm during fertilization [1].

A possible role for insects or leaches in mechanical transmission of the virus has been suggested [68,71,100,141]. Mechanical transmission has been artificially simulated by studies that have used injection (e.g., intramuscular, intracoelomic) as a means of infecting study animals (e.g., [72]), although ingestion of an infected insect by an amphibian also is possible. Regardless, an invertebrate would likely be acting as a fomite and serve in mechanical transmission of the virus [1]. The role of invertebrates in ranavirus transmission needs further investigation.

## 4. Conservation

### 4.1. Anthropogenic Stressors

Anthropogenically disturbed environments can increase the likelihood of ranavirus epizootics. In a survey of ponds containing green frogs in Ontario, Canada, industrial activity, human habitation, and degree of human influence (e.g., distance to nearest road) had significant effects on ranavirus presence, whereas other (natural) variables showed no correlation [38]. Two studies also suggest cattle grazing can increase the likelihood and severity of ranavirus epizootics [66,128]. Probability of infection by ranavirus in green frog tadpoles was 3.9× greater in farm ponds with cattle compared to those where cattle were excluded [66]. Elevated ammonia, which is known to stress amphibian larvae, may have contributed to greater infection rates in cattle ponds [66]. Emergent vegetation density also was lower at the cattle-access ponds in this study [142], which can contribute to greater larval clustering and pathogen transmission [128].

Environmental contaminants have also been shown to increase amphibian susceptibility to ranavirus infections. Environmental contamination may influence disease emergence in many ways, including disrupting homeostasis and compromising the host immune system [143,144]. A number of recent studies have investigated the effects of ecologically relevant doses of common pesticides on ATV infections in tiger salamanders. In long-toed salamanders (*A. macrodactylum*), ATV resulted in lower mortality at intermediate levels of the widely used herbicide atrazine [88]. However, in tiger salamanders, intermediate and high levels of atrazine decreased leukocyte counts and increased infection rates at intermediate levels [145]. Thus, atrazine could result in increased infection and disease emergence in some salamander populations. When combined with atrazine, the heavily-used insecticide chlorpyrifos had an additive effect, increasing susceptibility to ATV infection and mortality relative to either stressor alone [146]. Carbaryl, the most commonly used household pesticide in North America, also showed an additive effect when combined with predation and increased mortality by 50% [88]. In addition to pesticides, one field study showed that aluminum was correlated with ranavirus outbreaks [47].

### 4.2. Captive Breeding and Amphibian Trade

A major concern involves anthropogenic movement and introduction of invasive, nonnative amphibian, fish and reptile species that may act as ranavirus carriers; a phenomenon called pathogen pollution. American bullfrogs are commonly infected with FV3 and FV3-like ranaviruses, and are thought to contribute to the spread of ranaviruses via commercial trade [80]. Moreover, ranavirus strains isolated from bullfrog colonies appear to be highly virulent [9,67]. These results are consistent with theory that suggests high pathogen virulence (*i.e.*, ability to cause disease) can evolve in captive environments where high host density facilitates transmission [147]. Schloegel *et al*. [148] reported that over 28 million amphibians were imported into the United States during 2000–2005, with an 8.5% prevalence of ranavirus infections. Inasmuch as American bullfrogs are traded globally, this species may contribute significantly to the international movement of ranaviruses [149].

Recent evidence also suggests long-distance movement of ATV by infected salamanders that are used as fishing bait [52,132]. Salamanders collected from bait shops have repeatedly tested positive for ATV infection at high prevalence, and the introduction by anglers of infected bait salamanders, either accidentally or intentionally, has resulted in the spread of ATV [52,53,110]. An experiment showed that a bait ATV strain was significantly more virulent than native strains [132]. These results raise concern that introduction of infected bait tiger salamanders may introduce novel and highly virulent viral strains into areas with naive hosts or into areas where hosts have been previously exposed, but are adapted to other ranavirus strains.

In general, it is likely that the environmental stress of captivity increases susceptibility to ranavirus infections, given that ranaculture and aquaculture operations are generally single-species facilities designed to maximize animal production. Various fish species have also been found to be positive for ranaviruses, and in aquaculture, ranavirus infection and disease has sometimes resulted in large-scale fish mortality events. Empirical evidence suggests that ranaviruses that infect fish can cause disease in amphibians, or *vice versa* [150]. Further, comparative genomic analyses of ranavirus strains suggest at least one host switch from fish to amphibians has occurred [123]. As such, the introduction of fish species to an area may present a serious disease threat to native amphibians, if proper precautions are not taken to ensure that they are ranavirus free.

As a result of the above concerns, diseases caused by the ranaviruses that infect amphibians are now listed as notifiable by the World Organization for Animal Health (OIE) Aquatic Animal Health Code [149], which sets the standards for testing animals transported across international borders. However, these policies have yet to be implemented. Prior to implementation, tests that can detect ranavirus with high specificity (and relatively low costs), minimum sample sizes, and non-destructive sampling techniques must be identified, along with approved diagnostic laboratories [93]. Testing protocols have been developed for Epizootic Haematopoietic Necrosis Virus, which is a reportable ranavirus that infects fish [151].

### 4.3. Management Options

Following the guidelines of the World Organization for Animal Health [152], regulations that require testing amphibians that are reared in captive facilities for commerce and have the potential to be released or escape into wild populations should be considered (e.g., American and Asian bullfrog farms) [68,80,148]. For amphibians that are traded for fish bait (e.g., tiger salamanders, [53]), regulations that prohibit use of amphibians entirely or, at a minimum, outside the watershed from where they were captured, should be considered. Because ranaviruses can remain viable outside of hosts for a considerable duration [126], they can be transported on sampling equipment, recreational gear and fomites. Thus, biologists, recreationists and natural resource managers should decontaminate surfaces that come in contact with water bodies that contain amphibians to stop the unnecessary spread of the pathogen (see [153]). Managing ranaviral disease in captive facilities is more straightforward than in natural populations. Isolation of positive individuals and disinfection of animal enclosures are important initial steps, but similar to wild populations, it is essential to minimize possible stressors and maintain proper biosafety procedures to prevent cross contamination [93]. Warm (e.g., >25 °C) and frequently filtered water, along with low host densities, may be good preventative strategies to minimize ranavirus outbreaks in captivity [93,154].

The potential for development of a *Ranavirus* vaccine is promising, particularly considering that Majji *et al*. [67] found that prior infection with a ranavirus led to enhanced immunity against subsequent exposure. While use of a vaccine might have limited field utility, it could be valuable in captive populations. Researchers in Japan have developed a vaccine for the red sea bream *Iridovirus* that has proven successful in preventing infection in red sea bream [155–157]. Although not yet commercially available, an oral formulation of the vaccine is being tested for use in fish [157]. This iridoviral vaccine may be a useful starting point for development of a *Ranavirus* vaccine.

## 5. Future Directions

On 8 July 2011, the First International Symposium on Ranaviruses was held in Minneapolis, Minnesota, USA at the Joint Meeting of Ichthyologists and Herpetologists that included presentations from 23 scientists from nine countries with expertise in biological sciences and veterinary medicine [158–160]. Post symposium discussions resulted in the following research recommendations. First, the group agreed there is a need to understand persistence of ranaviruses in host systems, specifically with regard to environmental persistence outside of the host [126], latent persistence within hosts via quiescent infections [112], and identification of host reservoirs (e.g., [121]).

The group also agreed that further immunological research is necessary. In particular, there is a need to expand our understanding of the routes of infection, target organs, and immune responses to ranavirus infection. Use of the African clawed frog model has produced considerable insight into host immune responses [96]; however, there is a need to expand research to include other amphibian species (e.g., [98]). Suggested model species in North America for controlled experiments included the wood frog (a highly susceptible species) and American bullfrog (a low susceptible species used in trade). Other model species were not discussed, but might include the common frog, midwife toad (*Alytes obstetricans*), and alpine newt (*Mesotriton alpestris cyreni*) for European experiments due to occurrence of these species in ranavirus die-offs.

Researchers also highlighted the need for future molecular work. Spatial genetic variation among ranaviruses has been documented [123,132] but we understand little as to whether genomic variation connotes enhanced ranavirus pathogenicity in certain amphibian populations. Candidate virulence genes have been identified based on homology with other viruses [161], but the function of several of these have yet to be confirmed. Gene knock-out or -down experiments can be very effective at identifying genes that contribute to virulence and immune evasion [162–166]. Comparison of ranavirus genomes isolated from amphibians, fish and reptiles also will help elucidate evolutionary host-shifts among ectothermic vertebrates [123,167]. Follow-up intraclass transmission experiments should be conducted when possible. Regarding diagnostics, sensitivity experiments using quantitative real-time PCR need to be performed (e.g., [168]), as well as development of loci other than the major capsid protein (which is highly conserved among ranaviral species) for specific diagnoses of infection.

Ranaviruses are emerging pathogens and a threat to global amphibian populations. We encourage researchers to work in multidisciplinary teams to identify factors contributing to ranavirus emergence and in formulating conservation strategies. Investigating factors at multiple levels of host organization (*i.e.*, cellular, organismal, population, and community) and across disciplines (e.g., immunology, microbiology, genetics, ecology, and veterinary science) is key to deciphering the complexities of the ranavirus-host system. The Global Ranavirus Consortium (GRC) is an excellent opportunity for scientists and veterinarians to interact and collaborate on ranavirus research [169]. In the future, the GRC will plan a second international symposium on ranaviruses, organize conference calls to discuss research directions and recent findings, and maintain a recent publications website. By working in teams and facilitating communication, the GRC hopes to encourage complementary research that addresses the most urgent research needs.

## Figures and Tables

**Table 1.** Global distribution of known cases of ranavirus infection and mortality in captive and wild amphibian populations.

**Continent**	**Nation**	**Family**	**Scientific Name**	**I,M^1^**	**W,C^2^**	**References**
Asia	China	*Cryptobranchidae*	*Andrias davidianus*	M	C	[11]
		*Ranidae*	*Rana dybowskii*	I	W	[12]
		*Ranidae*	*Rana grylio*	M	C	[13,14]
		*Ranidae*	*Rana tigrina*	M	C	[15]
	Japan	*Hynobiidae*	*Hynobius nebulosus*	M	C	[16]
		*Ranidae*	*Lithobates catesbeianus*	M	W	[17]
	Thailand	*Ranidae*	*Rana tigrina*	M	C	[18]
Australia	Australia	*Myobatrachidae*	*Limnodynastes ornatus*	M	C	[19]
Europe	Belgium	*Salamandridae*	*Tylototriton kweichowensis*	M	C	[20]
	Croatia	*Ranidae*	*Pelophylax esculenta*	M	W	[21]
	Denmark	*Ranidae*	*Pelophylax esculenta*	M	W	[22]
	Israel	*Bufonidae*	*Pseudepidalea viridis*	I	W	[23]
	Netherlands	*Ranidae*	*Pelophylax spp.*	M	W	[24]
		*Salamandridae*	*Lissotriton vulgaris*	M	W	[24]
	Portugal	*Salamandridae*	*Triturus marmoratus*	M	W	[25]
		*Salamandridae*	*Triturus boscai*	M	W	[25]
	Spain	*Alytidae*	*Alytes obstetricans*	M	W	[26]
		*Salamandridae*	*Ichthyosaura alpestris*	M	W	[27]
	Switzerland	*Ranidae*	*Pelophylax esculenta*	M	C	[28]
			*Pelophylax ridibundus*	M	C	[28]
	UK	*Alytidae*	*Alytes obstetricans*	M	W	[29]
		*Bufonidae*	*Bufo bufo*	M	W	[29,30]
		*Ranidae*	*Rana temporaria*	M	W	[2,6,29,31,32]
		*Salamandridae*	*Lissotriton vulgaris*	I	W	[29]
North America	Canada	*Ambystomatidae*	*Ambystoma mavortium*	M	W	[33–35]
			*Ambystoma* spp.	I	W	[36]
		*Hylidae*	*Hyla versicolor*	I	W	[36]
			*Pseudacris crucifer*	M	W	[37]
			*Pseudacris* spp.	I	W	[36]
		*Ranidae*	*Lithobates clamitans*	I, M	W	[37,38,39]
			*Lithobates pipiens*	I, M	W, C	[4,39,40–42]
			*Lithobates sylvaticus*	I, M	W	[7,36,37,39,40,43]
		*Salamandridae*	*Notophthalmus viridescens*	I	W	[36]
	Costa Rica	*Bufonidae*	*Bufo marinus*	I	C	[44]
	USA	*Ambystomatidae*	*Ambystoma jeffersonianum*	M	W	[45]
			*Ambystoma macrodactylum*	M	W	[45]
			*Ambystoma maculatum*	I, M	W	[3,46–49]
			*Ambystoma mavortium*	I, M	W	[50–54]
			*Ambystoma opacum*	M	W	[49]
			*Ambystoma tigrium*	I, M	W, C	[3,55]
		*Bufonidae*	*Anaxyrus americanus*	I	W	[55]
			*Anaxyrus boreas boreas*	I, M	W, C	[56,57]
		*Cryptobranchidae*	*Cryptobranchus alleganiensis alleganiensis*	I	W	[58]
		*Dendrobatidae*	*Dendrobates auratus*	I	C	[59]
			*Phyllobates terribilis*	I	C	[59]
		*Hylidae*	*Acris crepitans*	I	W	[55]
			*Hyla chrysoscelis*	I, M	W, C	[45,60]
			*Hyla cinerea*	M	W	[61]
			*Pseudacris clarkii*	M	W	[62]
			*Pseudacris crucifer*	M	W	[3,47,49]
			*Pseudacris feriarum*	I, M	W	[49,55]
			*Pseudacris regilla*	M	W	[45]
			*Pseudacris sierra*	M	W	[63]
		*Plethodontidae*	*Desmognathus conanti*	I	W	[64]
			*Desmognathus fuscus*	I	W	[65]
			*Desmognathus imitator*	I	W	[64]
			*Desmognathus monticola*	I	W	[65,64]
			*Dessmognathus ocoee*	I	W	[64]
			*Desmognathus quadramaculatus*	I	W	[65,64]
			*Desmognathus santeetlah*	I	W	[64]
			*Desmognathus wrighti*	I	W	[64]
			*Eurycea cirrigera*	I	W	[65]
			*Eurycea longicauda*	I	W	[65]
			*Eurycea lucifuga*	I	W	[65]
			*Eurycea wilderae*	I	W	[64]
			*Gyrinophilus porphyriticus*	I	W	[64]
			*Plethodon glutinosus* complex	I	W	[65]
			*Plethodon jordani*	I	W	[64]
		*Ranidae*	*Lithobates blairi*	M	W	[45]
			*Lithobates catesbeianus*	I, M	W, C	[3,47,65–70]
			*Lithobates clamitans*	I, M	W	[3,47,66,69,71]
			*Lithobates palustris*	I, M	W	[3,55,65]
			*Lithobates pipiens*	I, M	W	[3,72–74]
			*Lithobates septentrionalis*	M	W	[3]
			*Lithobates sphenocephalus*	I, M	W	[45,55,71]
			*Lithobates sylvaticus*	I, M	W	[3,46–49,74,75]
			*Pyxicephalus adspersus*	M	C	[59]
			*Rana aurora*	M	W	[76]
			*Rana draytonii*	M	W	[45]
			*Rana heckscheri*	M	W	[45]
			*Rana luteiventris*	M	W	[56,61,63]
			*Rana muscosa*	M	W	[56]
		*Rhacophoridae*	*Rhacophorus dennysi*	M	C	[59]
		*Salamandridae*	*Notophthalmus viridescens*	I, M	W	[3,73,77]
		*Scaphiopodidae*	*Scaphiopus holbrookii*	I, M	W	[78]
South America	Argentina	*Leptodactylidae*	*Atelognathus patagonicus*	M	W	[79]
	Brazil	*Ranidae*	*Lithobates catesbeianus*	M	C	[80]
	Uruguay	*Ranidae*	*Lithobates catesbeianus*	I	C	[81]
	Venezuela	*Bufonidae*	*Bufo marinus*	I	W	[82]
		*Leptodactylidae*	*Leptodactylus* sp.	I	W	[82]

1 I = infection with no gross signs of ranaviral disease, M = mortality due to ranaviral disease.

2 W = wild population, C = captivity including zoological and ranaculture facilities; captivity does not include controlled virus challenges.
